# Hydrogen Sulfide Alleviates Skeletal Muscle Fibrosis via Attenuating Inflammation and Oxidative Stress

**DOI:** 10.3389/fphys.2020.533690

**Published:** 2020-09-18

**Authors:** Linlin Zhao, Xiaoguang Liu, Jing Zhang, Gaoyang Dong, Weihua Xiao, Xin Xu

**Affiliations:** School of Kinesiology, Shanghai University of Sport, Shanghai, China

**Keywords:** skeletal muscle, contusion, inflammation, fibrosis, hydrogen sulfide

## Abstract

The purpose of this study was to investigate the effect of exogenous hydrogen sulfide (H_2_S) treatment on skeletal muscle contusion. We established a skeletal muscle contusion model (S group) and an H_2_S treated of skeletal muscle contusion model (H_2_S group). Gastrocnemius muscles (GMs) were collected at day 1, day 5, day 10, and day 15 after injury, and comprehensive morphological and genetic analyses was conducted. H_2_S treatment reduced M1 macrophage (CD68), profibrotic cytokines (TGF-β), pro-inflammatory cytokines (TNF-α, IFN-γ, IL-1β, and IL-6), chemokines (CCL2, CCR2, CCL3, CCL5, CXCL12, and CXCR4), matrix metalloproteinases (MMP-1, MMP-2, MMP-9, and MMP-14) and oxidative stress factor (gp91phox) expression levels, improved M2 macrophage (CD206) level. Thus, exogenous H_2_S treatment reduced inflammation and oxidative stress, attenuated skeletal muscle fibrosis, and partly improved skeletal muscle injury.

## Introduction

Skeletal muscle injury is common in physical exercise (Tidball, 2011), and is treated with conservative regimens such as resting, ice, compression and elevation (Wright-Carpenter et al., 2004). Regeneration after skeletal muscle injury generally includes three stages: the first stage is the injury stage, which appears in the 1–3 days of the beginning of skeletal muscle injury, showing local swelling of the injured skeletal muscle, obvious hematoma, necrosis of some muscle tissue and inflammatory reaction. The second stage is the repair period, which generally occurs 5–10 days after skeletal muscle injury. Necrotic muscle tissue is swallowed and accompanied by regeneration of injured skeletal muscle. The third stage is the shaping stage of muscle tissue, which usually begins at 2–3 weeks after skeletal muscle injury, when the regenerated muscle fibers mature and accompany the formation of scar tissue (Xiao et al., 2016). In the initial phase after skeletal muscle injury, immediate treatment is key to a full repair outcome (Hardy et al., 2016). However, there is currently no effective strategy for treating skeletal muscle injury; due to the insufficient repair of muscle injury, complications such as muscle fibrosis and skeletal muscle dysfunction often occur (Xiao et al., 2016).

Hydrogen sulfide (H_2_S) has been considered an environmentally toxic gas for many years, but recent studies have found that H_2_S makes up a third class of gas signaling molecules, along with nitric oxide and carbon monoxide (Wang et al., 2019). As a metabolic, inflammatory, neurologic and vascular modulator, H_2_S plays an anti-hypertensive, anti-inflammatory and antioxidant role in the body (Veeranki and Tyagi, 2015). Recently, extensive studies have investigated the relationship between H_2_S and diseases. A growing amount of evidence suggests that supplemental H_2_S, especially in multiple disease models such as diabetes, hypertension, atherosclerosis, and heart/renal ischemia/reperfusion injury, can reduce oxidative stress, reduce levels of proinflammatory cytokine circulation interleukin-1β (IL-1β) and Chemokine (C-C motif) ligand 2 (CCL2), and regulate cellular redox balance, thereby reducing disease risk (Yang et al., 2011). In particular, H_2_S can significantly improve multiorgan ischemia-reperfusion injury (Yin et al., 2013). Furthermore, H_2_S have been studied in skeletal muscle biology and skeletal muscle diseases. NaHS treatment can improve hyperhomocysteinemia-mediated muscle injury by reducing oxicstress and reducing upregulation of muscle atrophy F-box (MAFBx) and muscle RING-finger protein-1 (MuR F-1) (Majumder et al., 2018), and Pharmacologic preconditioning with NaHS confers significant longterm protection against ischemia-reperfusion injury (IRI) in skeletal muscle for ischemic intervals (Henderson et al., 2011).

Here, a skeletal muscle contusion mouse model was established, to investigate the effect of exogenous H_2_S on inflammation and fibrosis in skeletal muscle contusion.

## Materials and Methods

### Mice

Eighty male ICR mice (age, 8 weeks; weight 36–40 g) were obtained from Shanghai Lab, Animal Research Center (Shanghai, China). Mice were housed in an environmentally controlled room with a constant temperature of 25°C, and ∼50% relative humidity, and a light-dark cycle of 12:12 h. Pellet food and water were provided *ad libitum*. Mice were randomly selected for the control group without muscle injury (Con group, *n* = 8). The muscle contusion group was intraperitoneally injected with saline (S group, *n* = 8 per group), and the H_2_S group was intraperitoneally treated with NaHS (Sigma-Aldrich, St. Louis, MO, United States) 28 μmol/kg each day for 15 days (H_2_S group, *n* = 8 per group) (Du et al., 2019). We used sodium pentobarbital anesthetized mice (Nembutal, 50 mg/kg body weight; intraperitoneal) during muscle injury induction. The left gastrocnemius muscle (GM) were used for morphological analyses, and the right GM were used for genetic analyses. The study was approved by the Ethics Review Committee for Animal Experimentation of Shanghai University of Sports (Shanghai, China) (reference number 2016006).

### Contusion Model

After mice were anesthetized with sodium pentobarbital, we fixed the hind leg of the mouse by extending the knee and dorsiflexing the ankle to 90°. A steel ball with a diameter of 15.9 mm (weight: 16.8 g) was dropped by a specific experimenter from a specific height (125 cm) through a tube with interior diameter of16 mm onto an impactor (Ota et al., 2011). The steel ball rested on the medial surface of the GM with a surface of 28.26 mm^2^. The muscle contusion caused a high-energy blunt injury. In this method the hit site of the GM in mice can immediately develop hematoma followed by marked skeletal muscle regeneration (Kasemkijwattana et al., 1998; Liu et al., 2018), which is very similar to the healing process in humans (Li et al., 2011). At different time points after injury (1, 5, 10, and 15 days), animals (*n* = 8) were sacrificed to harvest the GM.

### Hematoxylin and Eosin Staining

At 1, 5, 10, and 15 days post-injury, the left GMs were fixed with 4% paraformaldehyde for 24 h at room temperature (six mice per group). After paraffin embedding, we used a microtome (Leica-EG 1160, Leica Microsystems GmbH, Wetzlar, Germany) to cut the specimens into 4 μm cross sections from the midbelly region of GMs. Hematoxylin and eosin (H&E) staining was used to stain muscle histology sections for morphological analysis. Using a 20X objective, images were captured for each muscle section (Labophot 2, Nikon Corporation, Tokyo, Japan).

### Masson’s Trichrome Staining

Masson’s trichrome staining (D206, Jiancheng Bioengineering Institute, Nanjing, China) is mainly used to distinguish muscle fibers from collagen fibers, which can reflect the degree of skeletal muscle fibrosis (Fukushima et al., 2001). The percentage of collagen fiber area relative to the total area was calculated by using ImageJ software (ImageJ 1.44, Bethesda, MD, United States) (six mice per group).

### Collagen Analysis

Sirius red (Sigma, St. Louis, MO, United States) was used to stain sections. We viewed these sections under polarized light microscopy to determine the birefringence patterns, which indicated the degree of collagen organization. Using ImageJ to calculate the areas of collagen fibers, Collagen I and Collagen III were identified as red to orange and green fibers, respectively (de Melo et al., 2016).

### RNA Extraction and cDNA Synthesis

Around 110 mg of the right GMs (*n* = 8 per group) was homogenized using an Ultra-Turrax homogenizer (IKA Group, Staufen, Germany) in a solution of TRIzol reagent (cat no. 15596018; Invitrogen; Thermo Fisher Scientific, Inc., Waltham, MA, United States). We used a modified guanidinium isothiocyanate-CsCl method to isolate total RNA (Chirgwin et al., 1979). We used a spectrophotometer to measure the absorbance at 260 and 280 nm, and the concentration and purity of the total RNA were determined (NanoDrop 2000, Thermo Fisher Scientific, Inc.). Used Revertaid First Strand cDNA Synthesis kit from Fermentas (cat. no. K1622; Thermo Fisher Scientifc, Inc.) to reverse-transcribed the total RNA into cDNA. cDNA was synthesized using 2 μg of total RNA, 0.2 μg of random primers, 20 mM dNTP mix, 5X reaction buffer, 20 units of RiboLock RNase inhibitor and 200 units of Revertaid M-MuLV reverse transcriptase in a total volume of 20 μl.

### Real-Time Polymerase Chain Reaction (PCR)

Ten microliters of 2X Maxima SYBR Green/ROX qPCR Master mix (Vazyme), 1 μL of cDNA, nuclease-free water and 300 nM of each primer (Table 1) were used to carry out quantitative PCR in triplicate. The threshold cycle (the number of cycles needed to reach the detection threshold) was determined for each reaction, and the levels of the target mRNAs were quantified relative to the level of the housekeeping gene GAPDH using the 2^–ΔΔ*Cq*^ method (Livak and Schmittgen, 2001).

**TABLE 1:** Primers used for qRT-PCR.

Target gene	Forward primer sequences	Reverse primer sequences
CD68	5′-CAAAGCTTCTGCTGTGGAAAT-3′	5′-GACTGGTCACGGTTGCAAG-3′
CD163	5′-GCAAAAACTGGCAGTGGG-3′	5′-GTCAAAATCACAGACGGAGC-3′
CD206	5′-GGATTGTGGAGCAGATGGAAG-3′	5′-CTTGAATGGAAATGCACAGAC-3′
Col1a1	5′-GAGCGGAGAGTACTGGATCG-3′	5′-GCTTCTTTTCCTTGGGGTTC-3′
Col3a1	5′-GTCCACGAGGTGACAAAGGT-3′	5′-GATGCCCACTTGTTCCATCT-3′
gp91phox	5′-CCAGTGAAGATGTGTTCAGCT-3′	5′-GCACAGCCAGTAGAAGTAGAT-3′
IL-1β	5′-TGACGTTCCCATTAGACAACTG-3′	5′-CCGTCTTTCATTACACAGGACA-3′
IL-6	5′-GAACAACGATGATGCACTTGC-3′	5′-CTTCATGTACTCCAGGTAGCTATGGT-3′
TNF-a	5′-CTTCTGTCTACTGAACTTCGGG-3′	5′-CACTTGGTGGTTTGCTACGAC-3′
INF-γ	5′-GCTTTGCAGCTCTTCCTCAT-3′	5′-GTC ACC ATCCTTTTGCCAGT-3′
CCL2	5′-GCTCAGCCAGATGCAGTTAAC-3′	5′-CTCTCTCTTGAGCTTGGTGAC-3′
CCL3	5′-ACCATGACACTCTGCAACCA-3′	5′-CCCAGGTCTCTTTGGAGTCA-3′
CCL5	5′-CATATGGCTCGGACACCA-3′	5′-ACACACTTGGCGGTTCCT-3′
CCR2	5′-GAAAAGCCAACTCCTTCATCAG-3′	5′-TCTAAGCACACCACTTCCTCTG-3′
CXCR4	5′-CAAGGCCCTCAAGACGACAG-3′	5′-CCCCCAAAAGGATGAAGGAG-3′
CXCL12	5′-ACGGAAGAACCAAAGAGAAAGA-3′	5′-CTCAGACAGCGAGGCACAT-3′
MMP-1	5′-AGTTGACAGGCTCCGAGAAA-3′	5′-CACATCAGGCACTCCACATC-3′
MMP-2	5′-ACCCTGGGAGAAGGACAAGT-3′	5′-ATCACTGCGACCAGTGTCTG-3′
MMP-9	5′-CGTCGTGATCCCCACTTACT-3′	5′-AACACACAGGGTTTGCCTTC-3′
MMP-14	5′-CCTGGCTCATGCCTACTTCC-3′	5′-GCACAGCCACCAAGAAGATG-3′
TGF-β	5′-TGCGCTTGCAGAGATTAAAA-3′	5′-CGTCAAAAGACAGCCACTCA-3′
GAPDH	5′-ACTCCACTCACGGCAAATTC-3′	5′-TCTCCATGGTGGTGAAGACA-3′

### Statistical Analysis

Data are presented as the mean ± the standard deviation (SD) and were analyzed by repeated-measure analysis (SPSS 20.0; IBM Corp., Armonk, NY, United States). *Post hoc* multiple comparisons were performed by using the Bonferroni test. We tested the scar tissue area and data were compared using an independent samples *t*-test. Statistical significance was set at *P* < 0.05.

## Results

### H_2_S Donor Ameliorates Skeletal Muscle Injury After Contusion

**FIGURE 1:**
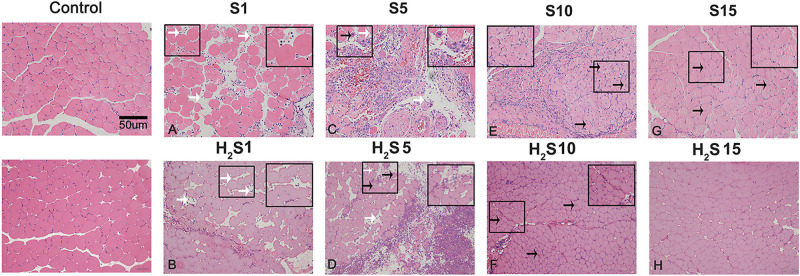
H_2_S donor ameliorates skeletal muscle injury after contusion. Control, uninjured skeletal muscle; S, muscle contusion group injected intraperitoneally with saline; H_2_S, muscle contusion group injected intraperitoneally with NaHS. Scale bars = 50 μm. White arrows indicate inflammatory cells; black arrows indicate central nucleation. **(A–H)** The muscle healing process in GM at 4 time points (1, 5, 10, and 15 days postinjury).

Skeletal muscle tissue was stained with H&E and observed under a 20-lens objective to evaluate whether H_2_S donor ameliorates skeletal muscle injury after contusion. H&E staining showed that, fewer inflammatory cells and significantly more central-nucleated regenerating muscle fibers were observed in the skeletal muscle from the H_2_S treated group than in the skeletal muscle from the muscle contusion group at 1 and 5 days post injury (Figures 1A–D). Additionally, H&E staining showed that the injured skeletal muscle from H_2_S treated mice exhibited improved morphology. At 10 and 15 days post-injury, there were still a few central-nucleated regenerating muscle fibers in the muscle contusion group, while the damaged area in the H_2_S group was almost entirely replaced by intact skeletal muscle fibers (Figures 1E–H).

### H_2_S Donor Attenuates Skeletal Muscle Fibrosis Caused by Contusion

**FIGURE 2:**
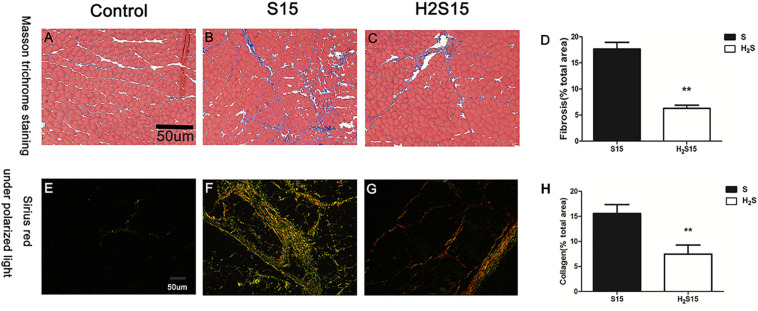
Representative images of Masson’s trichrome staining and Sirius red staining of injure GM at 15 days. **(A–C)** Masson’s trichrome staining; **(D)** Quantification of the scar tissue area in injured skeletal muscle; **(E–G)** Sirius Red with polarized light; **(H)** Quantification of collagen fibers in injured skeletal muscle. Scale bars = 50 μm. All data represent means ± SD, ^∗∗^Significant difference between S15 and H_2_S15, ^∗∗^*P* < 0.01, *n* = 6.

**FIGURE 3:**
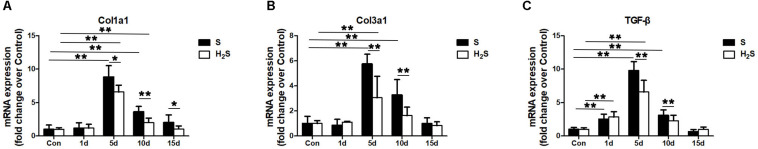
Effect of H_2_S donor on the expression of Collagen and pro-fibrotic cytokines in the GM post-injury. Control, uninjured skeletal muscle; S, muscle contusion group injected intraperitoneally with saline; H_2_S, muscle contusion group injected intraperitoneally with NaHS. **(A)** The expression of Collagen I; **(B)** The expression of Collagen III; **(C)** The expression of TGF-β. All data represent means ± SD, ^∗^*P* < 0.05, ^∗∗^*P* < 0.01, *n* = 8.

Collagen fibers were quantitatively analyzed by Masson staining and Sirius red staining. NaHS treatment at a dose of 28 μmol/kg for 15 days markedly attenuated collagen deposition in muscle contusion mice (17.65 ± 5.30 vs 5.45 ± 2.09, 15.58 ± 6.17 vs 7.27 ± 2.65, *P* < 0.01) (Figure 2). Moreover, we measured the mRNA expression levels of Collagen I and III with RT-PCR. The data showed that compared with the muscle contusion group, the H_2_S treatment group had significantly reduced the mRNA expression of Collagen I at 5 (*P* < 0.05), 10 (*P* < 0.01), and 15 days (*P* < 0.05) after injury (Figure 3A). The expression of Collagen III mRNA was also significantly reduced 10 and 15 days after contusion injury in the H_2_S treatment group compared with that in the muscle contusion group (*P* < 0.01) (Figure 3B). Transforming growth factor-β (TGF-β) is one of the most potent profibrotic cytokines, the expression of TGF-β mRNA was also significantly reduced 5 and 10 days after contusion injury in the H_2_S treatment group compared with that in the muscle contusion group (*P* < 0.01) (Figure 3C).

### Effect of H_2_S Donor on the Expression of Macrophage Specific Markers in Contused Muscle

**FIGURE 4:**
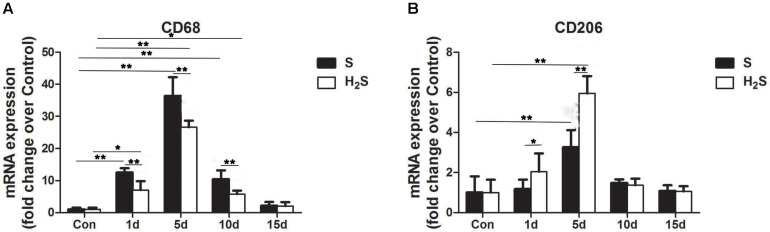
Effect of H_2_S donor on the expression of Macrophages in the GM post-injury. Control, uninjured skeletal muscle; S, muscle contusion group injected intraperitoneally with saline; H_2_S, muscle contusion group injected intraperitoneally with NaHS. **(A)** The expression of CD68; **(B)** The expression of CD206. All data represent means ± SD, ^∗^*P* < 0.05, ^∗∗^*P* < 0.01, *n* = 8.

CD68 and CD206 are specific markers of M1 macrophages and M2 macrophages, respectively. The RT-PCR data showed that the mRNA expression of the M1 macrophage marker CD68 significantly increased at 1, 5, and 10 days post-injury (*P* < 0.01). In addition, the expression of CD68 mRNA decreased 1 (*P* < 0.05), 5 (*P* < 0.01), and 10 days (*P* < 0.05) post-injury in the H_2_S donor group (Figure 4A). The H_2_S donor-treated group had significantly higher mRNA expression of CD206, a molecular marker of M2 macrophages at 1 day (*P* < 0.05) and 5 day (*P* < 0.01) post-injury than the muscle contusion group (Figure 4B).

### H_2_S Donor Decreased the Expression of Inflammatory Cytokine Levels in Contused Muscle

**FIGURE 5:**
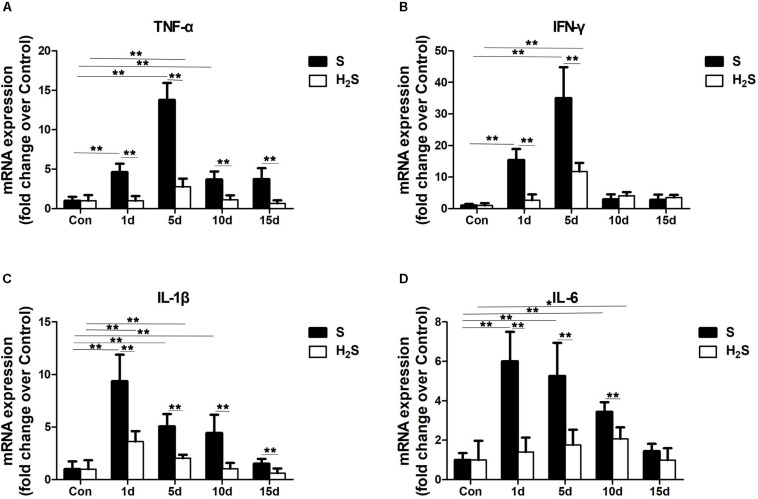
Effect of H_2_S donor on the expression of pro-inflammatory cytokines in the GM post-injury. Control, uninjured skeletal muscle; S, muscle contusion group injected intraperitoneally with saline; H_2_S, muscle contusion group injected intraperitoneally with NaHS. **(A)** The expression of TNF-α; **(B)** The expression of IFN-γ; **(C)** The expression of IL-1β; **(D)** The expression of IL-6. All data represent means ± SD, ^∗^*P* < 0.05, ^∗∗^*P* < 0.01, *n* = 8.

We measured the mRNA expression of pro-inflammatory cytokines (TNF-α, IFN-γ, IL-1β, and IL-6). The data showed that these pro-inflammatory cytokines all peaked (*P* < 0.01) in the early stage of regeneration post-injury. The H_2_S donor significantly decreased the expression of these pro-inflammatory cytokines (such as TNF-α, IFN-γ, IL-1β, and IL-6) (*P* < 0.01), indicating that H_2_S treatment attenuated the inflammatory response in the regeneration of contused muscle (Figure 5).

### H_2_S Donor Decreased the Expression of Chemokine Levels in Contused Muscle

**FIGURE 6:**
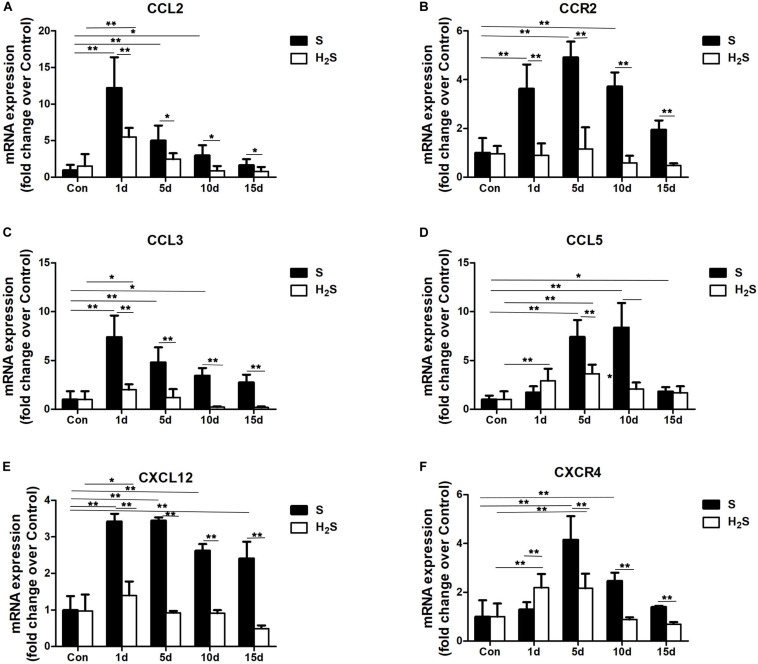
Effect of H_2_S donor on the expression of Chemokines in the GM post-injury. Control, uninjured skeletal muscle; S, muscle contusion group injected intraperitoneally with saline; H_2_S, muscle contusion group injected intraperitoneally with NaHS. **(A)** The expression of CCL2; **(B)** The expression of CCR2; **(C)** The expression of CCL3; **(D)** The expression of CCL5; **(E)** The expression of CXCL12 **(F)** The expression of CXCR4. All data represent means ± SD, ^∗^*P* < 0.05, ^∗∗^*P* < 0.01, *n* = 8.

Chemokines involved in the chemotaxis of immune cells were tested to gain insight into the mechanism of skeletal muscle regeneration after H_2_S therapy. Compared with the uninjured control group, the data showed that the expression of CCL2, CCR2, CCL3, CCL5, and CXCL12 significantly increased at 1, 5, and 10 days (*P* < 0.05; *P* < 0.01) post-injury. CXCR4, the receptor of CCL12 significantly increased at 5 and 10 days (*P* < 0.01). Except for CCL5 and CXCL12, the above chemokines returned to normal levels at 15 days post-injury. The H_2_S donor group had decreased expression of CCL2, CCR2, CCL3, CCL5, CXCL12, and CXCR4 throughout the regeneration stage compared with the muscle contusion group (Figures 6A–F).

### H_2_S Donor Decreased the Expression of Matrix Metalloproteinases in Contused Muscle

**FIGURE 7:**
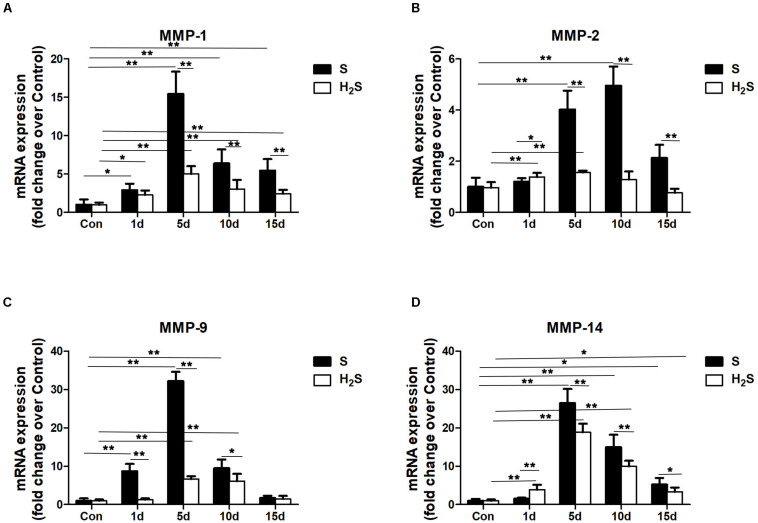
Effect of H_2_S donor on the expression of matrix metalloproteinases in the GM post-injury. Control, uninjured skeletal muscle; S, muscle contusion group injected intraperitoneally with saline; H_2_S, muscle contusion group injected intraperitoneally with NaHS. **(A)** The expression of MMP-1; **(B)** The expression of MMP-2; **(C)** The expression of MMP-9; **(D)** The expression of MMP-14. All data represent means ± SD, ^∗^*P* < 0.05, ^∗∗^*P* < 0.01, *n* = 8.

We tested the mRNA expression of matrix metalloproteinases (MMPs) (i.e., MMP-1, MMP-2, MMP-9, and MMP-14). The data showed that MMP-1, MMP-2, MMP-9, and MMP-14 mRNA significantly increased in injured skeletal muscle. There was a significantly decrease in MMP-1, MMP-2, MMP-9, and MMP-14 at the late stage of regeneration in the muscle contusion group compared with the H_2_S treatment group (*P*<0.05; *P* < 0.01) (Figures 7A–D).

### H_2_S Donor Decreased the Expression of Nicotinamide Adenine Dinucleotide Phosphate (NADPH) Oxidase in Contused Muscle

**FIGURE 8:**
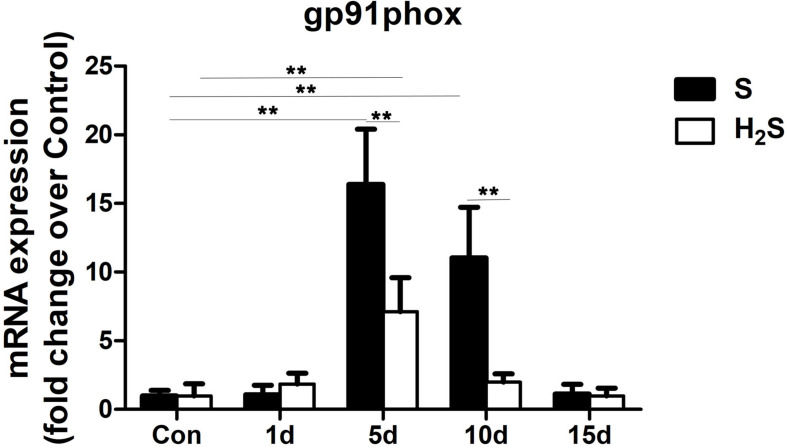
Effect of H_2_S donor on the expression of gp91phox in the GM post-injury. Control, uninjured skeletal muscle; S, muscle contusion group injected intraperitoneally with saline; H_2_S, muscle contusion group injected intraperitoneally with NaHS. All data represent means ± SD, ^∗^*P* < 0.05, ^∗∗^*P* < 0.01, *n* = 8.

As a key subunit of NADPH oxidases., Gp91phox was measured in our study. The results of the present study demonstrated that gp91phox mRNA levels significantly increased at 5 and 10 days after skeletal muscle injury (*P* < 0.01). However, H_2_S treatment significantly decreased the expression of gp91phox at 5 and 10 days post-injury (*P* < 0.01) (Figure 8).

## Discussion

We used heavy objects to strike the GM of mice to establish a muscle blunt contusion model, and we used a skeletal muscle contusion model that was treated with H_2_S, investigate the protective effect of exogenous H_2_S on skeletal muscle injury repair and the related mechanisms. H&E staining showed that cexogenous H_2_S treatment improved skeletal muscle injury repair (Figure 1). In addition, the results of Masson staining and Sirius red staining showed that gastrocnemius fibrosis was significantly reduced in the H_2_S-treated mice group than did in the muscle contusion mice group at 15 days (Figure 2). In various tissue damage models, the damage repair process is dysregulated, and extracellular matrix (ECM) deposition gradually increases, resulting in fibrosis, which manifests as a large increase in Collagen I and Collagen III, TGF-β as one of the most effective pro-fibrotic cytokines, its circulation level will also increase (Serrano and Munoz-Canoves, 2010; Liu et al., 2019a). In a rat model of peritoneal fibrosis, H_2_S treatment reduced type III collagen and alleviated fibrosis (Lu et al., 2015). Similar experimental results were also presented in this study. H_2_S treatment reduced the expression of Collagen I at 5, 10, and 15 days after skeletal muscle injury, decreased the expression of Collagen III and TGF-β at 5 and 10 days after injury. These findings suggest that H_2_S therapy improves skeletal muscle injury repair and reduces muscle fibrosis after contusion. This result has been observed in models of myocardial fibrosis (Huang et al., 2012), pulmonary fibrosis (Du et al., 2019), renal fibrosis (Song et al., 2014), and cirrhosis (Tan et al., 2011).

Skeletal muscle contusion is usually accompccalized by a local inflammatory response (Xiao et al., 2016). The intensity of the inflammatory response and the degree of tissue damage can be reflected by the expression level of these inflammatory factors (Zhu et al., 2015). There is evidence that H_2_S has an inhibitory effect on inflammation in various fibrosis-related diseases (Lu et al., 2015). IL-1β is secreted by many tissues and acts both locally and systemically. The overexpression of the IL-1β receptor or exposure to IL-1β increases the progression of atherosclerosis, whereas knockdown of IL-1β or inhibition of IL-1β reduces the biomarkers and lesions associated with atherosclerosis in animal studies. Treatment with HS inhibits IL-1β levels in atherosclerotic cell experiments, slowing progress in metabolic syndrome and atherosclerosis (Jain et al., 2010). Jain et al. noted that, blood H2S levels are significantly lower in fasting blood obtained from type two diabetes patients compared with age-matched healthy subjects, and in streptozotocin-treated diabetic rats compared with control Sprague–Dawley rats. They further prove that supplementation with H2S or an endogenous precursor of H2S (l-cysteine) in culture medium prevents IL-8 and MCP-1 secretion in high-glucose–treated human U937 monocytes (Jain et al., 2014). To evaluate the effect of exogenous H_2_S on skeletal muscle-damaging inflammation, we examined the expression of macrophage-specific markers. M1 macrophages can secrete a variety of pro-inflammatory factors, aggravating the inflammatory response; however, M2 macrophages can reduce the inflammatory response and secrete a variety of muscle regeneration regulators, which are related to the regeneration of muscle fibers (Magatti et al., 2017). In this study, compared with the skeletal muscle injury group, H_2_S treatment group had reduced mRNA levels of an M1 macrophage (CD68) post-injury (Figure 4A) and increased mRNA levels of an M2 macrophage (CD206) (Figure 4B). This was the same as the expected result, because previous models of renal injury (Du et al., 2019) and myocardial infarction (Qipshidze et al., 2012) showed that after H_2_S supplementation, M2 macrophage expression levels increased, and a corresponding recovery of renal and myocardial function occurred. In addition, we also examined the mRNA expression levels of inflammatory cytokines after muscle injury. TNF-α, IFN-γ, IL-1β, and IL-6 are important cellular inflammatory factors. The results of this study showed that the mRNA expression levels of TNF-α, IL-1β, and IL-6 significantly increased 1, 5, and 10 days after injury in the skeletal muscle contusion group (Figures 5A,C,D), and a significant increase in IFN-γ mRNA levels on 1 and 5 days after injury (Figure 5B). The mRNA levels of inflammatory cytokines (TNF-α, IFN-γ, IL-1β, and IL-6) were significantly reduced in muscle-contused mice on 1 day of exogenous H_2_S treatment, and H_2_S continued to inhibit the expression of inflammatory cytokines during subsequent treatment (Figure 5). These results are similar to those from *in vivo* and *in vitro* studies of an ischemic injury model of the hind limb; specifically, the level of inflammatory cytokines (TNF-α, IL-1β, and IL-6) was significantly reduced 3 h after H_2_S treatment, compared with that of the ischemic injury group (Henderson et al., 2011). In addition, in a model of damage to intestinal epithelial barrier function, exogenous H_2_S plays a protective role by inhibiting TNF-α and IFN-γ(Chen et al., 2015). Taken together, these results suggest that H_2_S therapy may drive M1 macrophages to transition to M2 macrophages and to reduce the expression of inflammatory cytokines.

Chemokines play an important role in the recruitment of monocytes, neutrophils, macrophages and lymphocytes. Previous studies have found that H_2_S donors reduce neutrophil recruitment in different models (Melo et al., 2019). Our previous studies also found that severe fibrosis is associated with the high expression of multiple chemokines in damaged skeletal muscle (Xiao et al., 2016). Therefore, we also studied muscle chemokines (CCL2, CCL3, CCL5, and CXCL12) and chemokine receptors (CCR2, and CXCR4). In this study, the mRNA expression of chemokines (CCL2, CCL3, CCL5, and CXCL12) and chemokine receptors (CCR2, and CXCR4) in skeletal muscle contusions significantly increased, while hydrogen sulfide treatment reduced the expression of chemokines (CCL2, CCL3, CCL5, and CXCL12) and chemokine receptors (CCR2, and CXCR4) in injured skeletal muscle (Figure 6). H_2_S treatment reduced the expression of chemokines in a mouse pain model (Melo et al., 2019), mouse atherosclerosis models (Zhang et al., 2012), and lung injury models (Li et al., 2013), and our results were similar. In addition, chemokines also play a part in the fibrotic process of various injury models, for example, theCXCL12-CXCR4 chemokine axis is involved in liver fibrosis (Qin et al., 2018), and CCL2 is involved in pulmonary fibrosis in lung injury (Li et al., 2013). In summary, H_2_S therapy may improve fibrosis in injured muscles by lowering chemokine levels.

In addition, the expression of MMPs in the GM of mice was measured. MMPs are zinc-dependent endopeptidases responsible for the degradation of the ECM. MMPs can selectively digest pathological ECM in a variety of disease or injury models, and the improper digestion of the ECM can lead to fibrosis in different tissues (Chen and Li, 2009; Liu et al., 2019a). The results of this study showed that MMP-1, MMP-2, MMP-9, and MMP-14 were significantly increased in mice in the contusion group, while the H_2_S treatment group had decreased mRNA levels of MMPs (MMP-1, MMP-2, MMP-9, and MMP-14) compared with the contusion group (Figure 7). In models of diabetes-induced kidney injury (Li et al., 2017) and myocardial fibrosis (Xiao et al., 2015), H_2_S reduced kidney injury and myocardial fibrosis by lowering the levels of MMPs. In addition, MMPs are significantly up-regulated in the dystrophic muscle of mdx mice, and the absence or inhibition of MMPs can significantly improve muscle structure and function, and reduce muscle fibrous necrosis in MDX mice (Kumar et al., 2010; Hindi et al., 2013). This study suggests that the moderating effect of H_2_S on skeletal muscle fibrosis may be partially related to the regulation of MMPs.

It is well known that H_2_S has antioxidant, anti-apoptotic and anti-inflammatory effects, which can protect tissues in various organs from damage (Du et al., 2019). Studies have shown that the inhibitory effect of H_2_S on lung fibrosis (Du et al., 2019) and myocardial fibrosis (Xiao et al., 2015) is at least partly due to reduced oxidative stress. In addition, reactive oxygen species (ROS) induced by NADPH oxidase are involved in skeletal muscle-damaging fibrosis (Liu et al., 2019b). Therefore, we examined gp91phox, which is a key subunit and common marker of NADPH oxidase. The results showed that, there was a significant decrease in the expression level of gp91phox in muscle contusion group compared to the H_2_S-treated group (Figure 8), suggesting that H_2_S may have reduced skeletal muscle fibrosis by inhibiting the expression of NADPH oxidase.

However, the study only rely on mRNA level assessed the effect of exogenous H_2_S reduce the injury of skeletal muscle. Where possible, protein content needs to be evaluated to determine the specific mechanism by which H_2_S can treat skeletal muscle injury. To demonstrate the beneficial effects of H_2_S, a pharmacological approach can be used by treating animals with CSE/CBS inhibitors prior to contusion and assessing possible deterioration.

The results of this study indicate that exogenous H_2_S therapy can reduce skeletal muscle injury, and may reduce skeletal muscle fibrosis by inhibiting the inflammatory response and reducing oxidative stress. Our study provides theoretical support for the treatment of skeletal muscle injury with H_2_S. However, the specific mechanism and clinical effect of H_2_S for the treatment of skeletal muscle injury require further research.

## Data Availability Statement

The raw data supporting the conclusions of this article will be made available by the authors, without undue reservation, to any qualified researcher.

## Ethics Statement

The animal study was reviewed and approved by the Ethics Review Committee for Animal Experimentation of the Shanghai University of Sport, Shanghai, China (reference number 2016006).

## Author Contributions

XX and WX designed this study and helped to draft the manuscript. LZ carried out the data analysis and drafted the manuscript. LZ, XL, JZ, and GD performed the histological staining and carried out the real time PCR. All authors have read and approved the final version of the manuscript, and agreed with the order of presentation of the authors.

## Conflict of Interest

The authors declare that the research was conducted in the absence of any commercial or financial relationships that could be construed as a potential conflict of interest.

## Abbreviations

CCL3: chemokine (C-C motif) ligand 3
CCL5: chemokine (C-C motif) ligand 5
CCL8: chemokine (C-C motif) ligand 8
CCR2: C-C chemokine receptor type 2
CXCL12: C-X -C motif chemokine 12
CXCR4: C-X -C chemokine receptor type 4
TNF-α: tumor necrosis factor- α
GMs: gastrocnemius muscles
H&E: hematoxylin and eosin
H2S: hydrogen sulfide
IFN-γ: interferon γ
IL-1 β: interleukin 1 β
IL-6: interleukin 6
MMP-1: metalloproteinase-1
MMP-14: metalloproteinase-14
MMP-2: metalloproteinase-2
MMP-9: metalloproteinase-9
NaHS: sodium hydrosulfide
TGF-β: transforming growth factor β
CCL2: Chemokine (C-C motif) ligand 2.
